# Supplementary figures and images for: Tauopathy-associated tau modifications selectively impact neurodegeneration and mitophagy in a novel C. elegans single-copy transgenic model
Source: Mol Neurodegener. 2020 Nov 9;15:65. doi: 10.1186/s13024-020-00410-7 (PMC7654055; doi:10.1186/s13024-020-00410-7)

Supplementary Figure 1

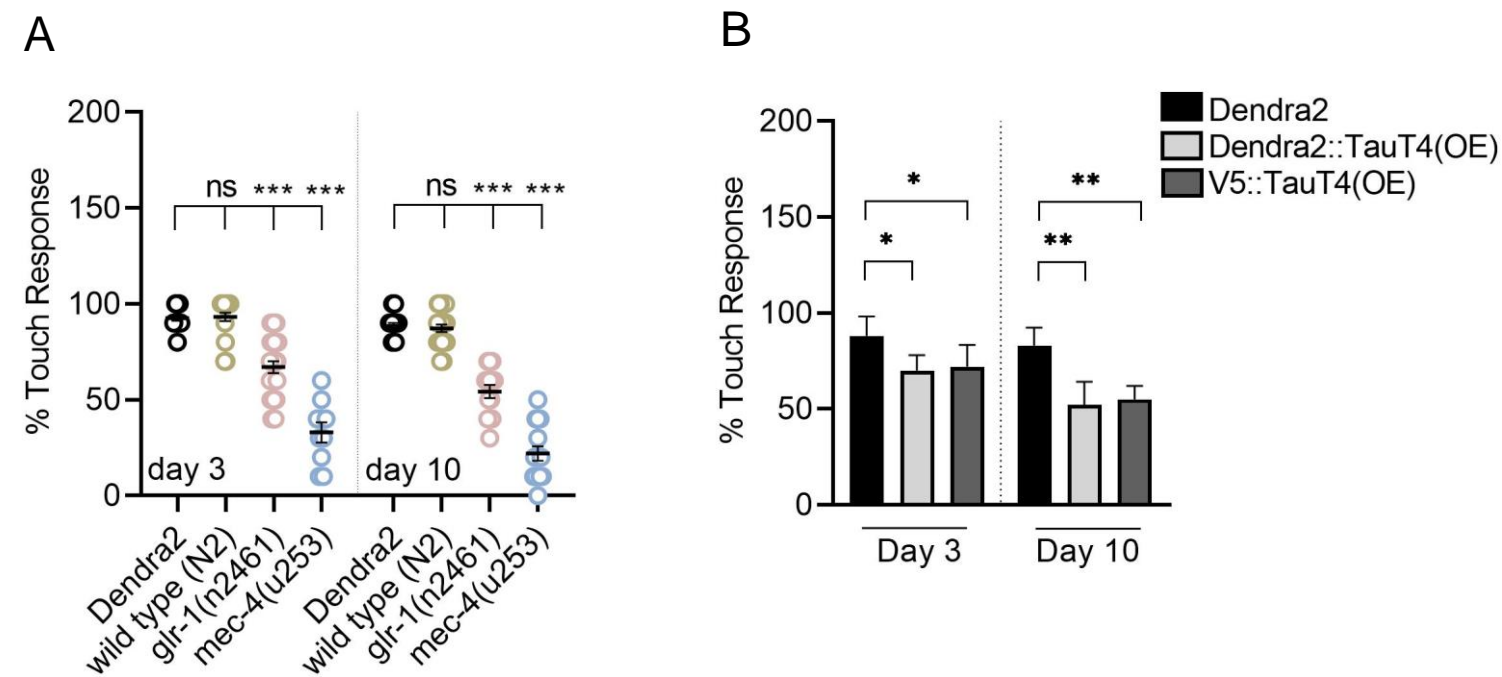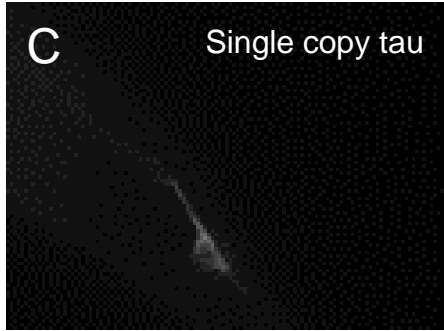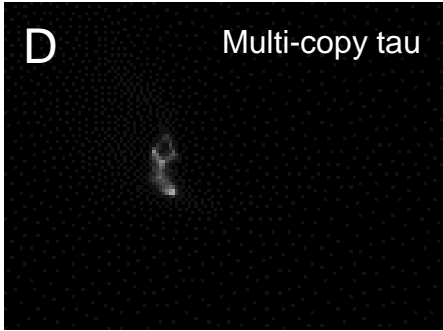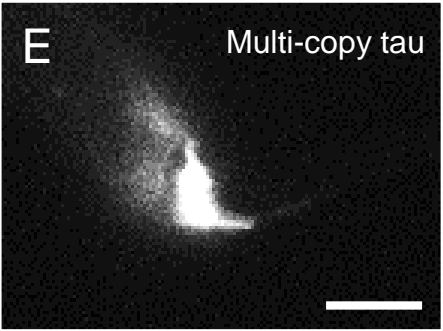

Supplement: Supplementary file 1 — Figure S1. Touch responsiveness controls and defects associated with tau overexpression. (A) Touch assays were conducted on day 3 and day 10 adult animals grown at 20 °C using glr-1(n2461) and mec-4(u253) strains as negative controls. The data are the mean ± SEM (N = 10–30 animals, from three independent replicates). Statistical analysis was by one-way ANOVA followed by Tukey’s multiple-comparisons test with *P < 0.001 denoting significance between bracketed samples (ns is not significant). Each point represents a value obtained from a single animal, which was touched 10 times, with a 10 s gap between each touch. (B) Bar graphs represent the average percent touch response of the overexpressed human tau, fused with Dendra2 or V5. Experiment was performed on day 3 and day 10 adult animals with N = 15–20. All the p-values are represented as *p < 0.05, **p < 0.01 and ***p < 0.001. Both data are from two biological replicates. (C-E) Representative images (40x magnification) of a PLM neuron in: (C) the MosSCI single-copy integrated tau strain (50 ms exposure), (D) a multi-copy array tau strain (5 ms exposure), and (E) a 50 ms exposure of the multi-copy strain, suggesting a 10-fold difference in relative expression level. (DOCX 367 kb) [file 13024_2020_410_MOESM1_ESM.pdf]

Supplementary Figure 2

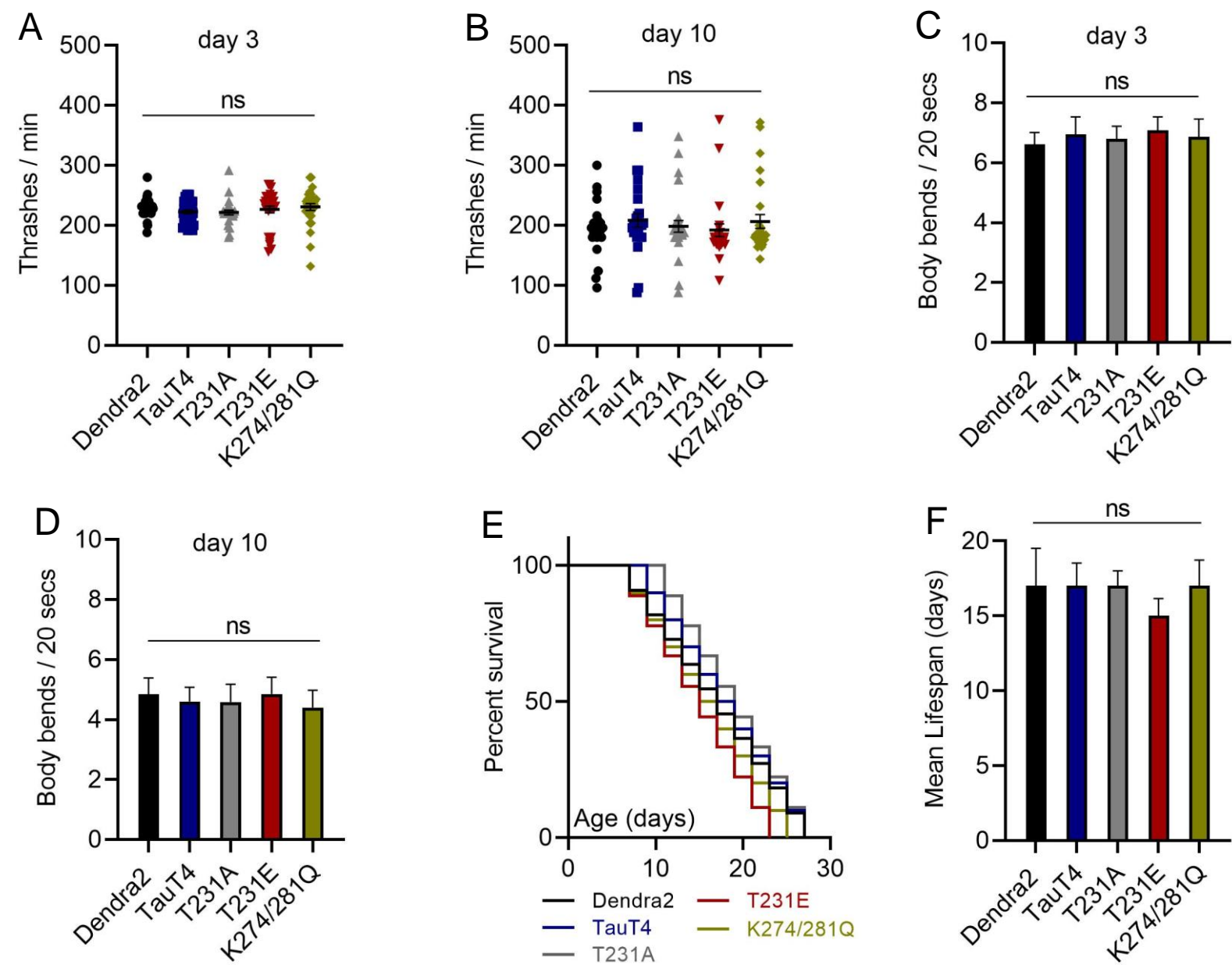

Supplement: Supplementary file 2 — Figure S2. Thrashing, locomotion, and lifespan are unaltered in strains with Tau mutations mimicking post-translational modifications to T231 and K274/281. Dendra2, TauT4, T231A, T231E and K274/281Q PTM mutant strains at day 3 and at day 10 of adulthood were assessed for (A, B) thrashing behavior in liquid media, (C, D) locomotion on solid media measured as the rate of body bends, (E) survival, or (F) lifespan. The data are the mean ± SEM. (N = 20-to-50 worms per genotype from three independent biological replicates, with each data point in A, B representing an individual worm). Statistical analyses were by one-way ANOVA or Mantel-Cox/ log-rank analysis. No statistical differences were found between genotypes for any of the three measures (denoted ns). (DOCX 313 kb) [file 13024_2020_410_MOESM2_ESM.pdf]

Supplementary Figure 3

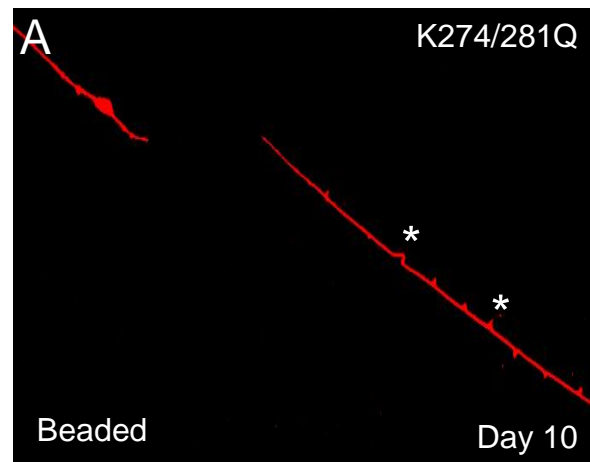

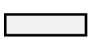 % worms with normal neuronal morphology

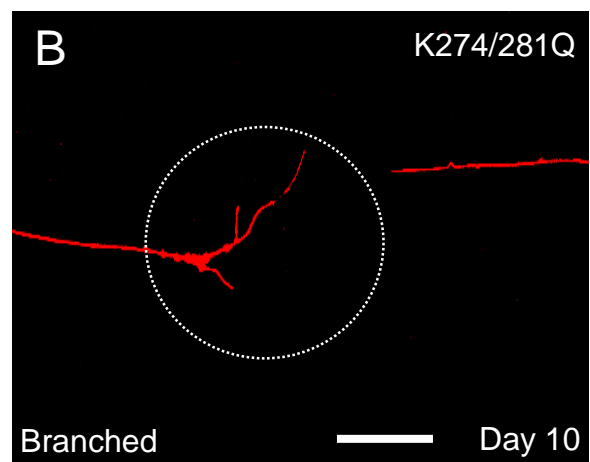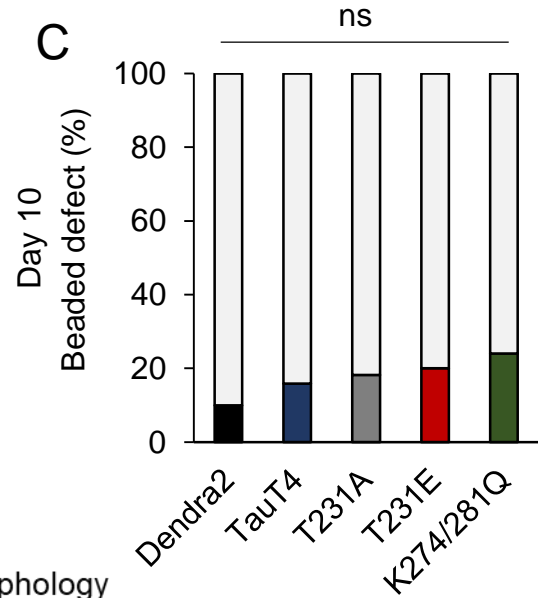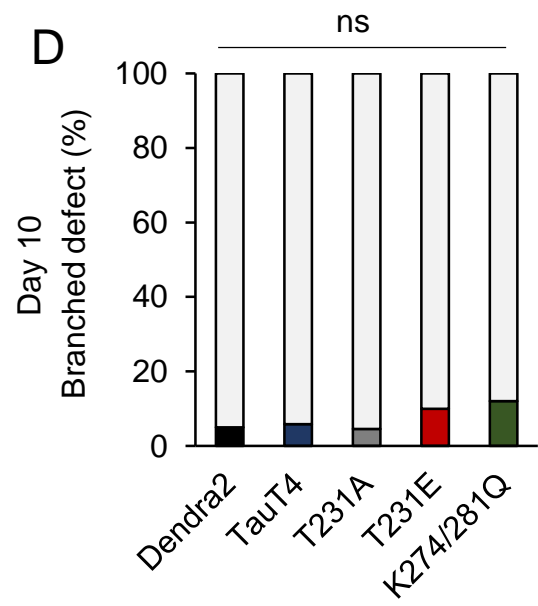

Supplement: Supplementary file 3 — Figure S3. Abnormal touch receptor neurite morphology in the older animals. Representative images of specific neurite morphology defects observed in touch cells, especially in day 10 adult animals. White stars illustrate beads or pearl like structures in panel A and dashed circle denote branching in panel B. The scale bar in panel B is 10 μm. (C, D) Quantification of the defects exemplified in panels A, B in Dendra2, TauT4, and T231A, T231E and K274/281Q. The colored bar denote the percentage of worms with the defect, while the gray bar denotes the percentage of worm that lack the defect. Statistical analysis was by Fisher’s exact test followed by two-tailed correction, with ns denotes statistically not significant. Data for the parental Pmec-4::mCherry reporter strain lacking tau transgenes, which is very similar to Dendra2, is not shown. N = 50 neurites from separate animals scored for each type of defect, from three independent biological replicates. (DOCX 143 kb) [file 13024_2020_410_MOESM3_ESM.pdf]

# Mito-mKeima: a biosensor for assessing mitophagy

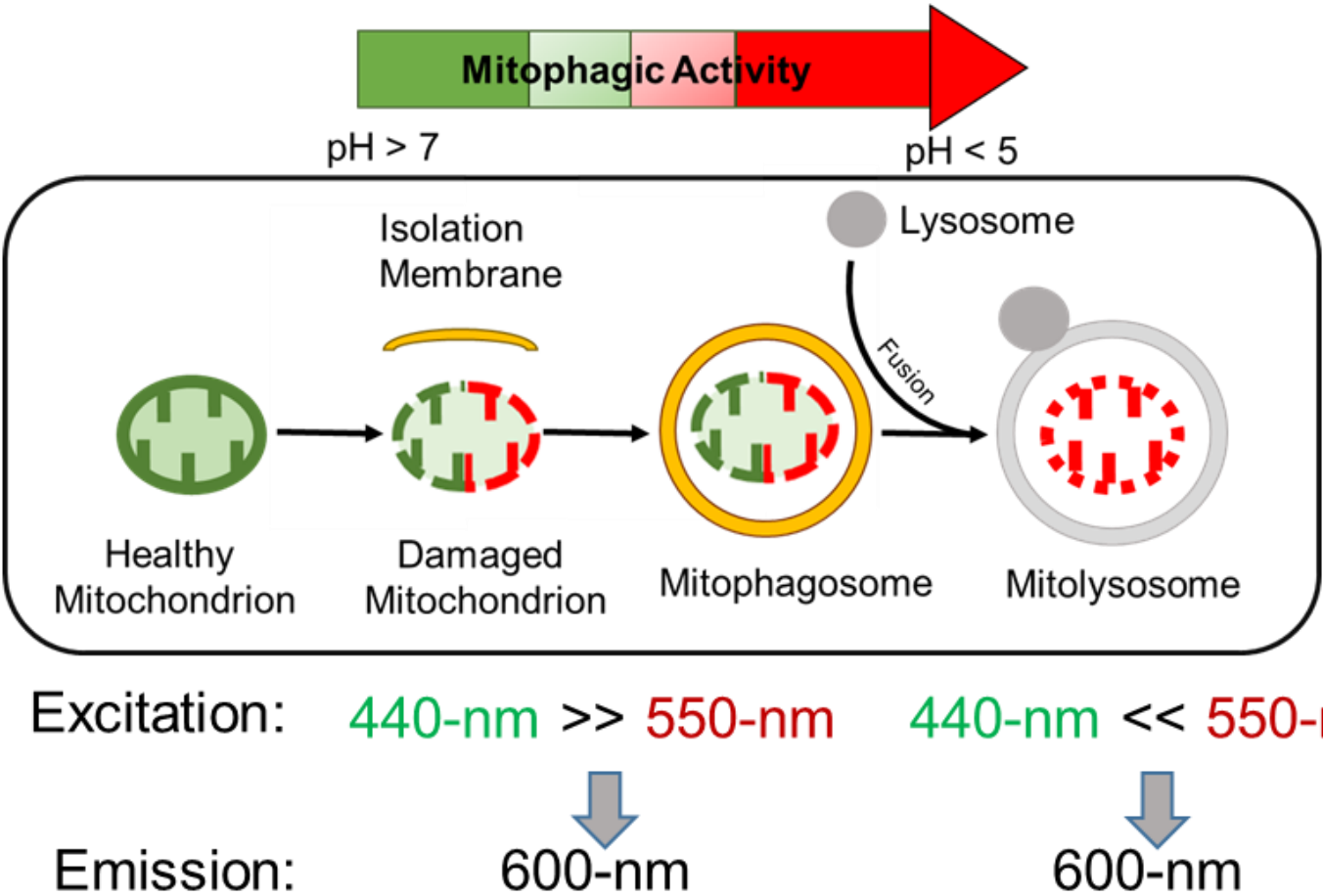

Supplement: Supplementary file 4 — Figure S4. Schematic representation of using mito-mKeima to measure mitophagy. Healthy mitochondria have a matrix pH > 7 (green) whereas dysfunctional mitochondria undergoing mitophagy and engulfed by lysosome are exposed to an acidic pH < 5 (red). Mito-mKeima is an acid-protease resistant fluorescent protein whose excitation maxima shifts from 440-nm to 550-nm with acidification. By measuring the ratio of emissions at 600-nm following sequential excitations at the two excitation maxima, you can obtain an estimate of the relative amount of mitochondria that are in neutral compared to acidic environments. In addition to spectral differences, mitochondria that have been engulfed by autophagosomes (labeled mitolysosomes) are generally round rather than tubuloreticular, and hence morphology can generally be used as a second measure to distinguish between organelles. However, this difference can be masked by mitochondrial fragmentation. (DOCX 140 kb) [file 13024_2020_410_MOESM4_ESM.pdf]

Supplementary Figure 5

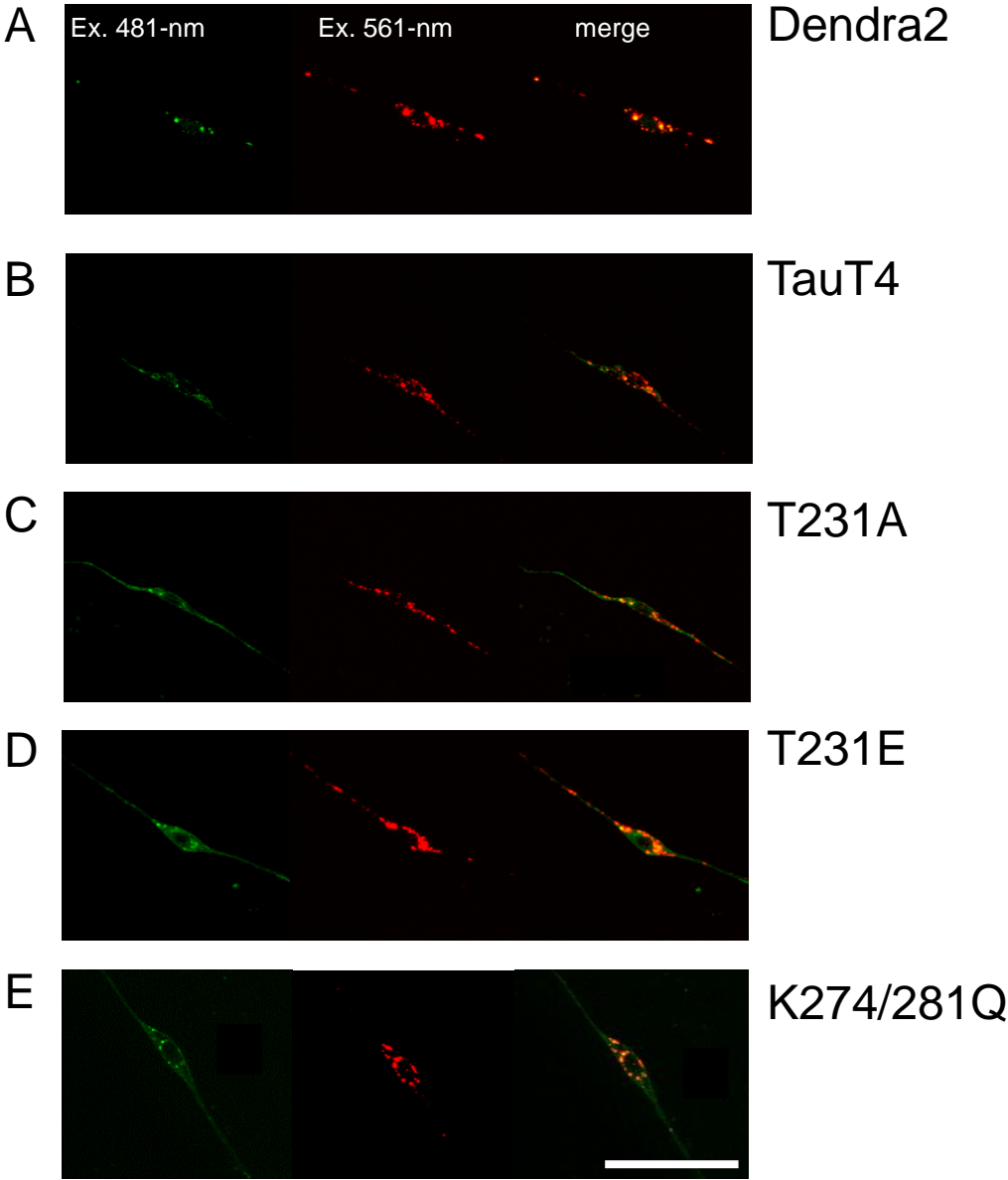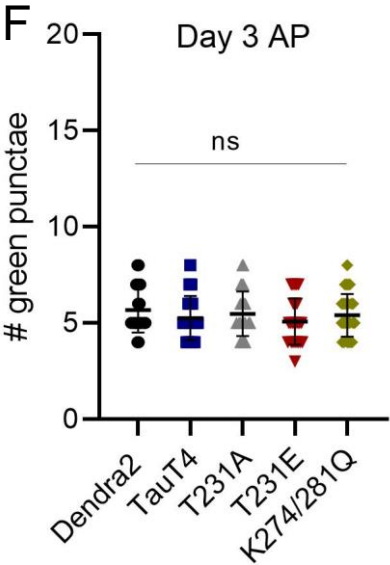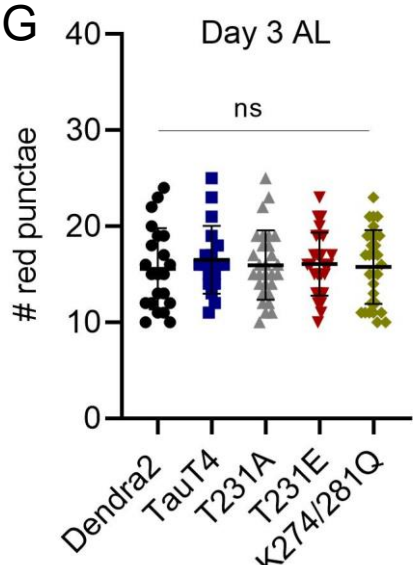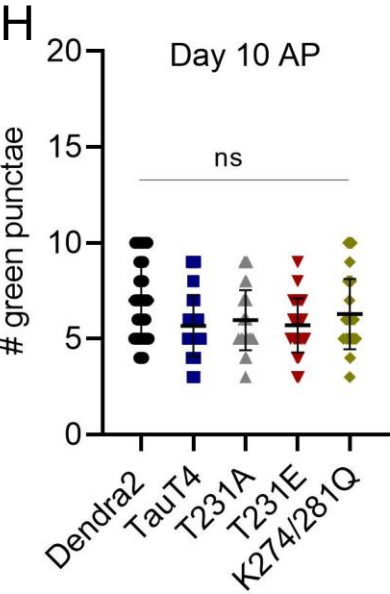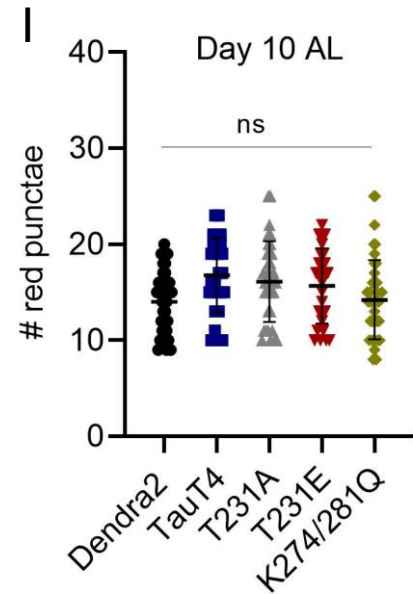

Supplement: Supplementary file 5 — Figure S5. Strains with tau mutations mimicking post-translational modifications to T231 and K274/281 have normal levels of baseline autophagy. (A-E) Representative fluorescent images from the PLM cell bodies expressing single-copy TauT4 or PTM-mimetics, together with an mCherry::gfp::lgg-1 reporter. Please note that the reporter fluorescence far exceeds and overwhelms that of the Dendra2 fusion. Autophagosome (AP) at neutral pH have been pseudo-colored green, and autolysosome (AL) have been pseudo-colored red. Scale bar = 100 μm. Note that the intensity of red fluorescence coming from distinct red AL compared to green of AP is stronger; thus the gain of red channel was purposefully set lower for all the images taken at Nikon Confocal microscope (see materials and methods) Quantification of APs and ALs in the PLM neurons of Day 3 (F, G) and Day 10 (H, I) Dendra2, TauT4, T231A, T231E and K274/281Q PTM mimetic strains. Data are the mean ± SD of ≥30 animals combined from two independent biological replicates. Data for the parental LGG-1 reporter strain lacking tau transgenes, which is very similar to Dendra2, is not shown. (DOCX 462 kb) [file 13024_2020_410_MOESM5_ESM.pdf]
