# Supplementary material for: Tauopathy-associated tau modifications selectively impact neurodegeneration and mitophagy in a novel C. elegans single-copy transgenic model
Source: Mol Neurodegener. 2020 Nov 9;15:65. doi: 10.1186/s13024-020-00410-7 (PMC7654055; doi:10.1186/s13024-020-00410-7)
Supplement: Supplementary file 6 — Table S1. Detailed quantification of the neuronal defects observed in day 10 transgenic worms. The most significant defects observed in the strains with tau mutations mimicking posttranslational modifications to T231 and K274/281 are the overextension of ALM/PLM and gaps or breaks observed in their neuronal processes. (DOCX 33.2 kb) [file 13024_2020_410_MOESM6_ESM.docx]

| **Transgene** | **Penetrance of defects in Day 10 animals** | | | | |
| --- | --- | --- | --- | --- | --- |
|  | Overextended | Misguided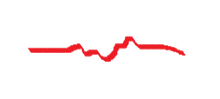 | Gap | Beaded | Branched |
| Dendra2 | 5 of 50 | 5 of 50 | 2 of 50 | 6 of 50 | 2 of 50 |
| TauT4 | 7 of 50 | 6 of 50 | 8 of 50 | 8 of 50 | 3 of 50 |
| T231A | 4 of 50 | 5 of 50 | 5 of 50 | 8 of 50 | 3 of 50 |
| T231E | 22 of 50 | 18 of 50 | 21 of 50 | 10 of 50 | 6 of 50 |
| K274/281Q | 21 of 50 | 9 of 50 | 21 of 50 | 13 of 50 | 7 of 50 |

**Table S1: Detailed quantification of the neuronal defects observed in day 10 transgenic worms.** The most significant defects observed in the strains with tau mutations mimicking posttranslational modifications to T231 and K274/281 are the overextension of ALM/PLM and gaps or breaks observed in their neuronal processes.
